# The Association of Bacterin and Recombinant Proteins Induces a Humoral Response in Sheep against Caseous Lymphadenitis

**DOI:** 10.3390/vaccines10091406

**Published:** 2022-08-27

**Authors:** Luan Santana Moreira, Natália da Rocha Lopes, Vitor Cordeiro Pereira, Caio Lopes Borges Andrade, Alex José Leite Torres, Marcos Borges Ribeiro, Songeli Menezes Freire, Ramon Mendes dos Santos, Milena D’ávila, Roberto Meyer Nascimento, Silvana Beutinger Marchioro

**Affiliations:** Laboratório de Imunologia e Biologia Molecular, Instituto de Ciências da Saúde, Universidade Federal da Bahia, Av. Reitor Miguel Calmon, Salvador 40231-300, BA, Brazil

**Keywords:** vaccine, recombinant protein, caseous lymphadenitis, immunization, *Corynebacterium pseudotuberculosis*

## Abstract

In this study, we investigated the capacity of the recombinant proteins SpaC, NanH, SodC, and PLD of *C. pseudotuberculosis* to trigger protective humoral and cellular immune responses against experimentally induced *C. pseudotuberculosis* infection in sheep. The antigens were produced in a heterologous system and were purified by affinity chromatography. Nine sheep were randomly divided into three groups, which were immunized as follows: Group 1 (control)—a mix of adjuvants composed of the inactivated T1 strain of *C. pseudotuberculosis* and commercial Montanide™ISA 61 VG (T1M); Group 2—rSpaC, rSodC, rPLD, and T1M; Group 3—rNanH, rSodC, rPLD, and T1M. All groups were immunized twice (on days 0 and 30) and challenged on day 90 of the experiment. Humoral and cellular immune responses were evaluated by Enzyme-Linked Immunosorbent Assay (ELISA) to quantify the IgG antibodies and interferon-gamma (IFN-y). Both vaccine formulations with recombinant proteins (groups 2 and 3) could induce a significant humoral IgG immune response in sheep. The proteins rSodC, rPLD, and rNanH were more immunogenic, inducing significant levels of IgG antibodies after the first dose of the vaccine or after the challenge, maintaining constant levels until the end of the experiment. However, it was not possible to differentiate between the cellular responses induced by the vaccines. This lack of effectiveness points toward the need for further studies to improve the efficacy of this subunit-based vaccine approach.

## 1. Introduction

*Corynebacterium pseudotuberculosis* is a Gram-positive bacterium that can survive and reproduce in macrophages. It can cause caseous lymphadenitis (CLA), which is characterized by the formation of granulomatous lesions, mainly in small ruminants [1]. This infection can manifest both superficially, being visible mainly in the pre-scapular, parotid, and mandibular region of the animal [2], and viscerally, appearing as lesions in several internal organs, such as the lungs, liver, kidneys, mediastinal lymph nodes, and sometimes in the mammary glands [3].

CLA is distributed worldwide in several regions and countries, where the livestock production of goats and sheep is among the major sources of income. In Brazil, CLA is endemic in the northeast region, reaching a prevalence of 97.14% in sheep [4]. Elsewhere, Minas Gerais in the southeast region also has a high prevalence of CLA [5,6]. The establishment of CLA in herds of sheep is directly associated with economic loss, due to the loss of animal products such as milk, wool, and even the carcass of the animal [7]. Although there is no way to fight *C. pseudotuberculosis* infections, herd immunization is among the most commonly employed strategies against the dissemination of the pathogen [8]. This mainly involves the discovery of new antigens that can modulate humoral and cellular immune responses [9].

The advancement of bioinformatics techniques has allowed the discovery of new antigens, facilitating the production of recombinant antigens that can be used for the production of novel vaccines. Since 2010, several studies have employed various recombinant proteins in different vaccine formulations offering varying levels of protection, such as phospholipase D (PLD) [10] and a *C. pseudotuberculosis* protein of 40 kDa (CP40) [11], as well as other antigens that are present in the *C. pseudotuberculosis* genome, such as neuraminidase H (NanH) and protein kinase G (PknG) [12]. These proteins, together with a putative adhesive pili tip protein (SpaC) and superoxide dismutase (SodC) are related to the virulence and pathogenicity of *C. pseudotuberculosis*, contributing to the level of interaction with the host’s cells or by evading its immune system. All these proteins were previously identified by bioinformatics analyses as promising proteins in the development of vaccines against CLA [13]. However, the majority of studies were conducted using murine models, which, despite being a crucial step in the process of evaluating new models or procedures, makes it difficult to extrapolate more precise information for the target animals in which CLA is established [14].

As the combination of recombinant antigens with inactivated bacteria may provide extra antigens and may elicit a broader range of protection in immunized animals [15], this study aimed to evaluate the immunomodulatory potential of four recombinant antigens (SpaC, NanH, SodC, and PLD) that are associated with the inactivated T1 strain of *C. pseudotuberculosis*, as well as the adjuvant Montanide™ ISA 61 VG (SEPPIC, Puteaux, France), in sheep that were experimentally infected with *C. pseudotuberculosis*.

## 2. Materials and Methods

### 2.1. Bacterial Strains and Culture Conditions

The bacterial strain *Escherichia coli BL21* Star (DE3) (Invitrogen, Carlsbad, California, USA) was used to produce the recombinant proteins. The strain was cultured in Luria–Bertani (LB) agar or broth (INVITROGEN, Carlsbad, California, USA) at 37 °C for approximately 16 h under agitation. Two strains of *C. pseudotuberculosis*, T1 and CapJ4, were used for the vaccine formulation and challenge, respectively. Both strains were cultured in brain-heart infusion (BHI) agar and broth (HIMEDIA, West Chester, PA, USA) at 37 °C for approximately 48 h.

### 2.2. Production of Recombinant Proteins

The entire gene codingfor *C. pseudotuberculosis* SpaC, NanH, SodC, and PLD was selected and then submitted for designing the synthetic versions, with codons optimized for expression in heterologous systems and then cloned in the commercial vector pD444-NH (DNA 2.0 Inc., Newark, CA, USA). This vector fuses six histidine residues to the heterologous protein, enabling its purification by affinity chromatography (Ni-Sepharose). The DE3 strain was transformed with the recombinant vectors by chemical and physical methods, following large-scale induction using 0.3 M isopropyl α-D-thiogalactoside (IPTG) (Sigma–Aldrich, Saint Louis, MO, USA) for 3 h. The medium was centrifuged for the aggregation of cells, resuspended in lysis buffer (0.2 M NaH_2_PO_4_, 0.5 M NaCl, 0.005 M imidazole) for 30 min, and sonicated 15 times with pulses of 15 s, then centrifuged for 1 h at 10,000× *g* at 4 °C, and finally solubilized in 8 M urea (rNanH, rPLD, and rSpaC) or 0.2% lauryl sarcosine (rSodC). The supernatants were collected and filtered through a 0.45 µm membrane. Then, the sample was purified by affinity chromatography using the Ni-Sepharose column, according to the manufacturer’s instructions (GE HEALTHCARE, Madison, WI, USA). The recombinant proteins were eluted in crescent concentrations of imidazole, dialyzed in PBS (pH 7.4), concentrated, and then quantified using Lowry’s method. The purity of the proteins was assessed by electrophoresing them in 12% SDS-PAGE.

### 2.3. Experimental Design

After receiving the approval of the Brazilian animal use ethics committee (protocol 1936220819), nine sheep of mixed races and at an age of 3monthsold were acquired and kept strictly under study conditions, in accordance with the ethical standards for the use of animals in research that have been established by the Brazilian legislation. Before the beginning of the experiment, all animals were tested to confirm the absence of antibodies against *C. pseudotuberculosis* through an indirect Enzyme-Linked Immunosorbent Assay (ELISA), using microplates sensitized with secreted antigens from *C.* *pseudotuberculosis* in BHI medium (as previously standardized in our laboratory). These animals were randomly assigned into three groups and immunized with the following vaccine formulations: Group 1 (negative control)—animals immunized with the inactivated T1 strain of *C. pseudotuberculosis* (10^6^ cells/mL) and Montanide™ ISA 61 VG (SEPPIC, Paris), a mineral-oil-based adjuvant (T1M); Group 2—animals immunized with the recombinant proteins rSpaC, rSodC, rPLD, and T1M; Group 3—animals immunized with the recombinant proteins rNanH, rSodC, rPLD, and T1M (see Table 1).

The animals were clinically examined before and after immunization, as well asmonitored daily by a trained team and weekly by a veterinarian, to assess their clinical status and well-being. The experiment lasted 240 days, during which 15 mL of whole-blood samples were collected via jugular vein puncture on days 0, 7, 14, 30, 45, 60, 75, 90, 105, 120, and 240.

On day 0, the animals were immunized with 100 µg of each respective antigen (rNanH, rPLD, rSodC, and rSpaC), 1 × 10^6^/mL of the T1 strain (inactivated by two cycles at a low temperature of −70 °C), and the commercial adjuvant Montanide™ ISA 61 VG in the proportions of 60% adjuvant and 40% antigen, with a final volume of approximately 800 µL of solution per animal. On day 30, a booster immunization was given. On the 90th day, the animals were challenged with 1 mL of inoculum containing 1 × 10^6^/mL of the biofilm-forming *C. pseudotuberculosis* strain CAPJ4, administered subcutaneously in the right periscapular region. This strain had previously been utilized in studies by our group [16]. The animals were euthanized on day 240.

### 2.4. Evaluation of the Humoral Response

Serum samples collected from the animals underwentan indirect ELISA assay for the detection of total IgG. For this assay, 96-well polystyrene plates (GREINER Bio-One, São Paulo, Brazil) were coated with the recombinant proteins at concentrations of 10 ng/well (rNanH and rSpaC), 50 ng/well (rSodC and rPLD; previously standardized), and 100 µL of *C. pseudotuberculosis-*secreted antigens in BHI medium [17], diluted in 0.05 M of carbonate/bicarbonate buffer (pH 9.6), and incubated overnight for 12 h in a humid chamber at 4 °C. The plates were washed with 0.01 M PBS containing 0.05% Tween 20 (PBS-T), blocked with 5% casein, and incubated for 2 h at 37 °C in a humid chamber. After washing with PBS-T, the sheep sera were diluted to 1:100 in PBS-T containing 1% casein and then incubated for 1 h at 37 °C in a humid chamber. Then, after further washing, secondary anti-sheep IgG antibodies conjugated with peroxidase enzyme and diluted to 1:20,000 were added and incubated for 45 min at 37 °C in a humid chamber. The reaction was marked with hydrogen peroxide and o-phenylenediaminedihydrochloride substrate (OPD) (Sigma–Aldrich, Saint Louis, MO, USA) diluted in phosphate citrate buffer for 10 min at room temperature and in the dark. The reaction was stopped by adding 25 µL of stop solution (2 M sulfuric acid), and then the absorbance was read using a spectrophotometer at 492 nm (THERMO PLACA, Miami, FL, USA).

### 2.5. Evaluation of the Cellular Immune Response

The interferon-gamma level in the blood cultures was quantified to measure the cellular immune response, using an Ovine IFN-y kit (MabTech AB, Nacka Strand, Sweden). The whole blood samples from animals, which were collected on days 0, 90, and 240, were incubated in a 24-well tissue culture plate (TPP, Switzerland) against various stimuli, such as negative control (culture medium), positive control (pokeweed—5 µg/mL), the *C. pseudotuberculosis* T1 strain, and the respective recombinant proteins from each group (10 µg/mL), for 24 h at 37 °C. After the incubation period, the cultures were centrifuged at 10,000× *g* for 15 min to separate the cells and the supernatant. The supernatant was used to quantify IFN-y according to the manufacturer’s protocol. Then, 96-well polystyrene plates were coated with 2 µg/mL of monoclonal antibody mAb MT17.1 solution, diluted in PBS (pH 7.4) and incubated overnight at 4 °C. The plates were then washed twice with PBS, blocked with a solubilization solution (PBS-T20 + 0.1% BSA), and incubated for 1 h at room temperature. After incubation, the plates were washed five times with PBS-T20, after which the IFN-y standard curve and samples were added at 100 µL per well, followed by incubation for 2 h at room temperature. This was followed by further washing with PBS-T20, after which the detection antibody MT307 (1:2000 dilution) was added at 100 µL per well and incubated for 1 h at room temperature. The plates were again washed with PBS-T20, after which streptavidin (at 1:1000 dilution) was added at 100 µL per well and incubated for another 1 h at room temperature. This was followed by a final washing with PBS-T 20. Then, the substrate tetramethylbenzidine (MBIOLOG Diagnósticos, Minas Gerais, Brazil) was added and incubated in the dark for 15 min; after the reaction was complete, it was stopped using a stop solution (2 M sulfuric acid). The plates were read in a spectrophotometer set at 450 nm.

### 2.6. Statistical Analysis

The samples were submitted for normality testing using the Shapiro–Wilk test and an analysis of repeated measures with mixed-effect models. The statistical program GraphPad Prism version 8.4.2 for Windows (GraphPad Software, San Diego, CA, USA) was used for the analysis. For normally distributed data, Holm–Sidak’s post hoc test was used, in which correlations were made between groups (Control–Group 2, Control–Group 3, and Group 2–Group 3). The *p*-values were <0.05.

## 3. Results

### 3.1. Recombinant Protein Production

**Figure 1:**
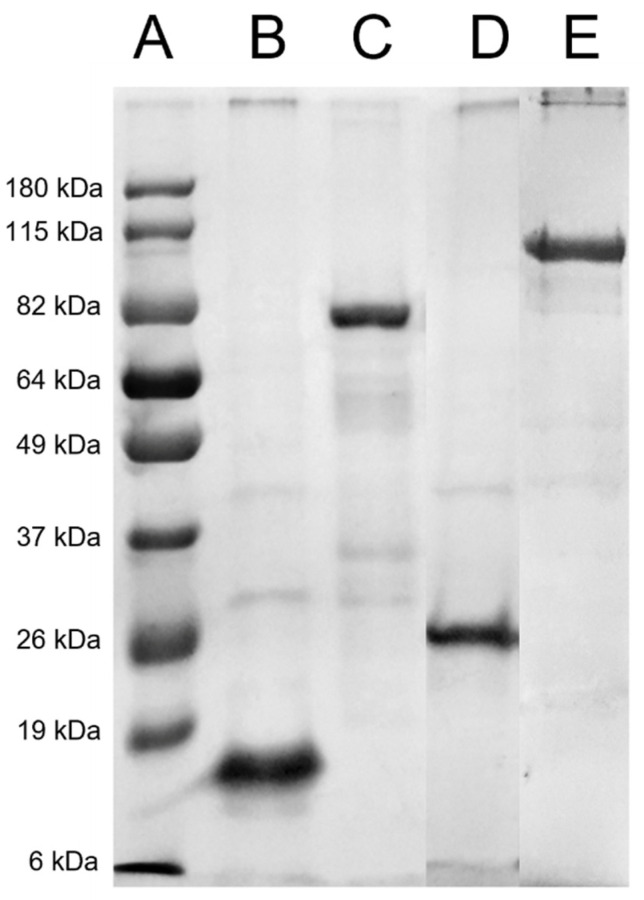
SDS-PAGE of purification at 12%. Recombinant proteins from *C. pseudotuberculosis* were produced in a heterologous system and stained with Coomassie blue. (**A**) Standard curve, (**B**) rSodC, (**C**) rNanH, (**D**) rPLD, and (**E**) rSpaC.

The purity of the recombinant proteins was evaluated using SDS-PAGE (Figure 1). The recombinant proteins rNanH (72 kDa), rPLD (32 kDa), and rSpaC(86 kDa) showed improved purity when solubilized in urea (8M), in contrast to the recombinant protein SodC (18 kDa), which showed better purity when solubilized in 0.2% lauryl sarcosine. The proteins were quantified using Lowry’s method; the determined concentrations were: rSodC = 3.62 mg/mL, rNanH = 3.22 mg/mL, rPLD = 2.32 mg/mL, and SpaC = 1.94 mg/mL.

### 3.2. Humoral Immune Response

**Figure 2:**
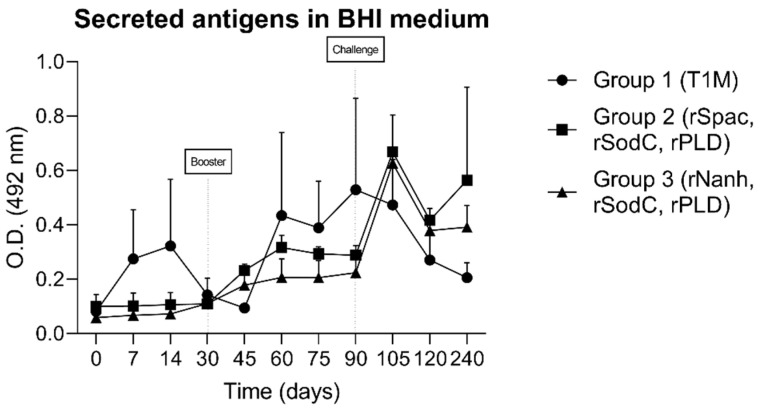
IgG responses from sheep serum immunized intramuscularly with bacterin and adjuvant T1M (group 1), rSpaC, rSodC, rPLD, T1M (group 2) and rNanH, rSodC, rPLD, T1M (group 3) at 0 and 30 days, as determined by an indirect ELISA. Microtiter plates were coated with secreted antigens of *Corynebacterium* *pseudotuberculosis* and the levels of IgG responses were determined on days 0, 7, 14, 30, 45, 60, 75, 90, 105, 120, and 240. Data represent the mean and standard deviation at OD 492 nm (*n* = 3) of sheep serum. All reactions were performed in duplicate.

The capacities of the recombinant proteins, which were co-administrated with the *C. pseudotuberculosis* bacterin, to induce serum IgG antibodies in sheep were evaluated by an ELISA assay. To assess the humoral response stimulated by the control group, i.e., Group 1 (vaccinated only with bacteria and the adjuvant), an indirect ELISA using the secreted antigens of *C. pseudotuberculosis* was performed parallel to the experiment, and the groups were compared statistically (Figure 2).

**Figure 3:**
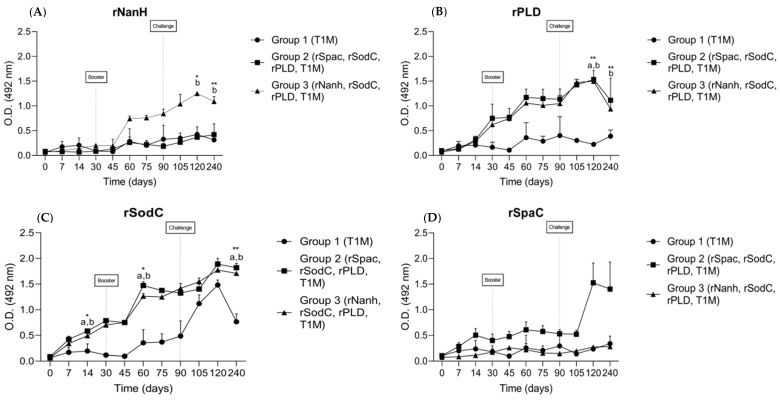
IgG responses from sheep serum immunized intramuscularly with bacterin and adjuvant T1M (group 1), rSpaC, rSodC, rPLD, T1M (group 2) and rNanH, rSodC, rPLD, T1M (group 3) at 0 and 30 days, as determined by an indirect ELISA. Microtiter plates were coated with recombinant proteins and the levels of anti-rNanH (**A**), rPLD (**B**), rSodC (**C**) and rSpaC (**D**). Responses were determined on days 0, 7, 14, 30, 45, 60, 75, 90, 105, 120 and 240. Data represent the mean and standard deviation OD 492 nm (*n* = 3) of sheep serum. All reactions were performed in duplicate. * *p* < 0.05; ** *p* < 0.01 compared with the sheep treated with recombinant proteins and bacterin (groups 2 and 3) and with the control group (group 1). Difference between groups was established by (a) statistical difference between group 2 and group 1 (control) and (b) statistical difference between group 3 and group 1 (control).

An indirect ELISA assay was also used to quantify the IgG antibodies produced by the recombinant proteins. Both groups receiving formulations containing co-administered recombinant proteins showed detectable antibodies, indicating that all proteins were immunogenic in sheep, although no statistical differences were observed between the groups. In addition, the control group that received only *C. pseudotuberculosis* bacterin along with the adjuvant only reacted significantly with the rSodC. The rNanH protein (Figure 3A) induced increased IgG production after the booster in group 3. After the challenge, the IgG levels continued to grow, reaching the highest levels on day 120 (*p* < 0.05), followed by a slight decrease in IgG production toward the end of the experimental period (*p* < 0.01). The recombinant protein rPLD (Figure 3B) induced IgG production in groups 2 and 3 before the second dose of immunization, with a significant difference from the control group after the challenge (*p* < 0.01), while the control group showed baseline levels of the antibody even after the challenge. The rSodC protein (Figure 3C) induced antibody production after the first dose of immunization in groups 2 and 3 (*p* < 0.05), with a continuous increase after the booster (*p* < 0.05) remaining stable until the challenge. One month after the challenge, groups 2 and 3 continued to show high levels of IgG, after which the levels remained stable and consistently greater compared to the control group until the end of the experiment (*p* < 0.01). Group 1 showed an increase in IgG production one month after the challenge, although this was in smaller amounts than groups 2 and 3, and then showed a declining pattern until the end of the experiment. The rSpaC protein (Figure 3D) was the only protein that failed to induce IgG production after the second dose of immunization. The levels of anti-rSpaC antibody showed an increased rate of production in group 2 only after the challenge, stabilizing until the end of the experiment.

### 3.3. Cellular Immune Response

**Figure 4:**
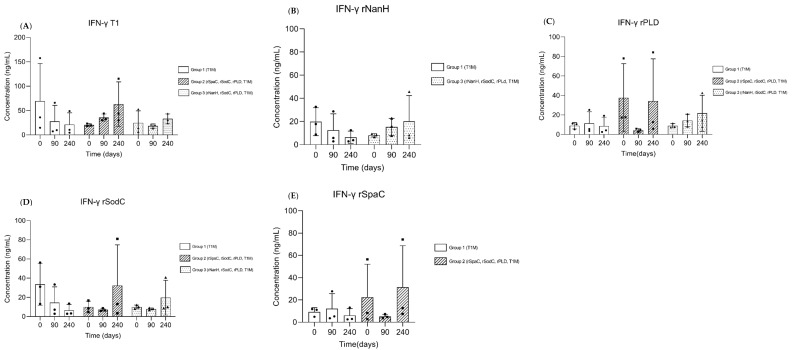
IFN-y response from sheep. Whole blood supernatant was stimulated with T1 (**A**) and recombinant proteins rNanh (**B**), rPLD (**C**), rSodC (**D**), and rSpaC (**E**), determined by anti-bovine IFN-γmAb MT307, employed as described in the manufacturer’s protocol. Responses were determined on days 0, 90, and 240. Data represent the mean and standard deviation at OD450(*n* = 3) of sheep serum supernatant.

To evaluate the level of induction of the cellular immune response, a sandwich ELISA was performed to quantify the levels of IFN-y in whole blood and the respective stimuli. Overall, we observed that the IFN-y levels increased after the challenge, both in the T1 stimulus and the recombinant proteins (Figure 4), although no significant differences between the groups were observed against any of the four antigens evaluated.

### 3.4. Post-Mortem Evaluation

After the experiment concluded, all the animals were euthanized, and the presence of superficial and visceral granulomatous lesions was evaluated. Macroscopic lesions were not observed in the internal organs of any of the animals. Two out of three animals from groups 1 and 3 presented a small superficial lesion near the challenge infection point. Only one animal from group 2 presented a granulomatous lesion in the left lymph node on the same side as that of the challenge. However, the severity of the lesions was the same in either the vaccinated (group 2 and 3) groups or the control group (group 1). This indicates that the experimental infection with the *C. pseudotuberculosis* strain was successful and that the infection could not be neutralized by the antibodies developed by the vaccines in these animals.

## 4. Discussion

The study investigated the capacity of the recombinant proteins SpaC, NanH, SodC, and PLD, antigens of *C. pseudotuberculosis*, for inducing protective humoral and cellular immune responses against experimentally induced *C. pseudotuberculosis* infection in sheep. Previous studies have demonstrated that these antigens are involved in host cell adhesion and invasion, nutrient acquisition, and evasion of the host immune system, which could play important roles in the development of vaccines against CLA [13,18].This study aimed to further improve the degree of protection by delivering pooled antigens, along with the inactivated T1 strain of *C. pseudotuberculosis* and the commercial adjuvant Montanide™ ISA 61 VG (SEPPIC, France). Two vaccines were formulated: rSpaC + rSodC + rPLD (group 2) and rNanH + rSodC + rPLD (group 3), both of which were associated with the adjuvant mix. Both formulations could induce a humoralIgG immune response in sheep. Some proteins, such as rSodC, rPLD, and rNanH, were more immunogenic once they had induced significant levels of IgG antibodies after the first dose of the vaccine or after the challenge.

Recombinant proteins are widely used in vaccine development, making them an effective tool in the research on novel vaccine formulations against CLA [14]. Moreover, recombinant vaccines offer several advantages compared to the traditional ones, which often have weak or poor immunogenicity and need an adjuvant for effective action [19]. It is well known that the selection of an adequate adjuvant is crucial to modulating the features of the antibody response that is induced by the vaccine, as well as to eliciting protection. In our trial, a bacterin produced by T1 (an attenuated strain of *C. pseudotuberculosis*), which could induce a strong cellular response in a previous study [15] after association with a commercial W/O emulsion (Montanide™ ISA 61 VG), was included as an adjuvant mix in our vaccine formulations. The association of bacterin and recombinant proteins is well established in the literature, indicating a high potential in inducing both humoral and cellular responses [20,21], as well as in passive immunity [22]. On the other hand, Montanide™ ISA 61 VG is a widely used adjuvant in the veterinary sector, demonstrating better results compared to aluminum-based adjuvants in cattle and sheep [23,24]. Unfortunately, we were not able to include a group that was vaccinated only with the recombinant antigens without the adjuvant, which could have helped measure the effect of the adjuvant in the evaluated formulations. However, considering the nature of vaccine formulation, we believe that the association of recombinant proteins with the adjuvant and bacterin may have influenced the induction of antibody production soon after the first immunization was given in our study. The dynamics during the production of IgG antibodies were determined against antigens secreted by *C. pseudotuberculosis*, as previously standardized in our lab [17]. Thus, we could evaluate the antibody production induced by the T1 strain that was present in the adjuvant control group. Although there were no statistical differences between groups immunized with formulations containing the recombinant proteins and the control group throughout the experimental period, IgG antibodies were detected in the control group, indicating that the attenuated T1 strain of *C. pseudotuberculosis* could induce a humoral response in these animals. The lack of statistical significance may be attributed to the complexity of the antigens of *C. pseudotuberculosis* that were used to coat the ELISA plates, as well as the small sample size of our experimental groups.

It is widely accepted that immunity against *C. pseudotuberculosis* infection involves both the humoral and cellular arms of the immune system [25]. In this study, we demonstrated that the IM application of the two experimental vaccine formulations (groups 2 and 3) induced a humoral IgG immune response in sheep that was statistically significantly higher (*p* < 0.05) compared to the control group (group 1). Of the four recombinant proteins evaluated, rSodC could induce the significant production of IgG antibodies soon after the first immunization, while rNanH and rPLD induced significant IgG production 30 days after the challenge. These results are in line with the literature, wherein mice immunized with rNanH showed increased production of the antibodies only from the second immunization, which yielded a survival rate of 60% after the mice were challenged with virulent *C. pseudotuberculosis* [12]. When we analyzed the longevity of these antibodies during the experimental period, which is important for vaccine development, we observed that the levels of IgG antibodies anti-rNanH and anti-rSodC remained constant until the end of the experimental period. This demonstrated that both proteins were good immunogens for constituting vaccine formulations against CLA. We also observed that the control group showed an increase in the production of anti-rSodC IgG after the challenge, indicating that SodC may be a constitutive protein among *C. pseudotuberculosis* strains. Although the role of SodC as a virulence factor of *C. pseudotuberculosis* has been recently studied, and high levels of expression in both abscess and culture samples have been reported [26], its immune-protective role against infection has not been backed up with sufficientin vivostudies.

Despite the induction of humoral immunity in vaccinated sheep, the evaluated vaccine formulations failed to induce a significant cellular immune response. The cellular immune response was evaluated through the quantification of IFN-y by a commercial assay kit. Stimuli from the individual recombinant proteins included in all vaccine formulations were used to identify how much each protein contributed to the production of IFN-y, as well as to the stimulation of the T1 strain. No statistical difference was observed between the groups. Nevertheless, we could identify a pattern in IFN-y production, wherein both groups that received the vaccine formulations containing the recombinant proteins (groups 2 and 3) showed an increase in the production of IFN-y after the challenge. This pattern was common in all groups subjected to immunization with the recombinant proteins, as well as the T1 stimulus. On the other hand, the control group (group 1) showed decreased production of this cytokine, which is an important biomarker required for full immuneprotection against *C. pseudotuberculosis* [27]. These results demonstrate the potential of the recombinant proteins to modulate a cellular immune response in our experimental groups. We hypothesize that two factors may have contributed to the lack of statistical significance between groups. Firstly, the small sample size of our experimental groups may have directly influenced the standard deviation, thus decreasing the statistical power. Secondly, variations between individual samples may have interfered with the first factor, although this phenomenon may be explained by the genetic variance between animals, which can influence the immune response more strongly [28].

Several studies have evaluated the recombinant proteins of *C. pseudotuberculosis* in various forms of administration and formulations. Some of them were evaluated individually as single antigens [12,29], while others were associated with attenuated bacteria [14], different adjuvants [30], or as a pool of antigens [31]. However, all these studies were performed in mice and cannot be extrapolated to the target species to determine the protective capacity of antigens against CLA. To the best of our knowledge, our study is the first to evaluate most of the recombinant proteins (rSodC, rNanH, and rSpaC) in the target species of CLA. Although no significant reduction of clinical signs was observed in this study, it must be noted that the challenge dose and route used in this model were extremely high and aggressive. This challenge model aimed to represent an exacerbated systemic infection. Thus, the level of protection offered by the vaccines formulated in this experimental challenge might be adequate to fight natural infection in the field.

## 5. Conclusions

The recombinant proteins administered intramuscularly in our vaccine formulations induced a humoral immune response in the sheep. However, the lack of effectiveness of such formulations in protecting against *C. pseudotuberculosis* infection suggests that further studies with a higher sample size are necessary. Moreover, to improve the efficacy of the vaccines against CLA, different antigens and adjuvant combinations that could improve protection against *C. pseudotuberculosis* infection in small ruminants should be tested, paving the way toward the development of a new generation of vaccines.

## Figures and Tables

**Table 1:** Experimental design of the different treatment groups. Nine animals were included in the study.

Group	N	D 0 ^b^	D 30	D 90	D 240
Group 1 (T1M ^a^)	3	First dose ^c^	Booster ^c^	Challenge infection	Euthanasia
Group 2 (rSpaC + rSodC + rPLD + T1M)	3	First dose	Booster	Challenge infection	Euthanasia
Group 3 (rNanH + rSodC + rPLD + T1M)	3	First dose	Booster	Challenge infection	Euthanasia

^a^ T1M-inactivated T1 strain of *C. pseudotuberculosis* (10^6^ cells/mL) and the Montanide™ ISA 61 VG (SEPPIC, Puteaux, France), a mineral-oil-based adjuvant. ^b^ D 0: Day of the experiment. ^c^ Application route: Intramuscular.

## Data Availability

Not applicable.

## References

[1] Zavoshti F.R., Khoojine A.B.S., Helan J.A., Hassanzadeh B., Heydari A.A. (2012). Frequency of caseous lymphadenitis (CLA) in sheep slaughtered in an abattoir in Tabriz: Comparison of bacterial culture and pathological study. Comp. Clin. Pathol..

[2] Chikhaoui M., Khoudja F.B. (2013). Clinico pathological investigation on caseous lymphadenitis in local breed sheep in Algeria. Trop. Anim. Health Prod..

[3] Jesse F.F.A., Odhah M.N., Abba Y., Garba B., Mahmood Z., Hambali I.U., Haron A.W., Lila M.-A.M., Zamri-Saad M. (2020). Responses of female reproductive hormones and histopathology in the reproductive organs and associated lymph nodes of Boer does challenged with Corynebacterium pseudotuberculosis and its immunogenic corynomycolic acid extract. Microb. Pathog..

[4] Alves J.R.A., de Farias A.E.M., dos Anjos D.M., Lima A.M.C., Faccioli-Martins P.Y., de Souza C.J.H., Pinheiro R.R., Alves F.S.F., de Azevedo S.S., Alves C.J. (2020). Seroepidemiological study of Caseous lymphadenitis in sheep from the Northeast region of Brazil using an indirect ELISA. Trop. Anim. Health Prod..

[5] Seyffert N., Guimarães A., Pacheco L., Portela R., Bastos B., Dorella F., Heinemann M., Lage A., Gouveia A., Meyer R. (2010). High seroprevalence of caseous lymphadenitis in Brazilian goat herds revealed by Corynebacterium pseudotuberculosis secreted proteins-based ELISA. Res. Vet. Sci..

[6] de Farias A.E.M., Alves J.R.A., Alves F.S.F., Pinheiro R.R., Faccioli-Martins P.Y., Lima A.M.C., de Azevedo S.S., Alves C.J. (2019). Seroepidemiological characterization and risk factors associated with seroconversion to Corynebacterium pseudotuberculosisin goats from Northeastern Brazil. Trop. Anim. Health Prod..

[7] Jordan D., Sentance C., Spooncer W., Balan J., Morris S. (2012). Inspection of lymph nodes for caseous lymphadenitis and its effect on the density of microbes on sheep carcasses. Meat Sci..

[8] Oreiby A.F., Hegazy Y.M., Osman S.A., Ghanem Y.M., Al-Gaabary M.H. (2014). Caseous lymphadenitis in small ruminants in Egypt: Clinical, epidemiological and prophylactic aspects. Tierärztl. Prax. Ausg. G GroßtiereNutztiere.

[9] Galvão C.E., Fragoso S.P., de Oliveira C.E., Forner O., Pereira R.R.B., Soares C.O., Rosinha G.M.S. (2017). Identification of new Corynebacterium pseudotuberculosis antigens by immunoscreening of gene expression library. BMC Microbiol..

[10] de Pinho R.B., Silva M.T.D.O., Brenner G., Alves M.S.D., Azevedo V., Portela R.D., Borsuk S. (2021). A novel approach for an immunogen against Corynebacterium pseudotuberculosis infection: An *Escherichia coli* bacterin expressing phospholipase D. Microb. Pathog..

[11] Droppa-Almeida D., Vivas W.L., Silva K.K.O., Rezende A.F., Simionatto S., Meyer R., Lima-Verde I.B., Delagostin O., Borsuk S., Padilha F.F. (2016). Recombinant CP40 from Corynebacterium pseudotuberculosis confers protection in mice after challenge with a virulent strain. Vaccine.

[12] Silva M.T.D.O., de Pinho R.B., Fonseca B.D.R., Bezerra F.S.B., Sousa F.S.S., Seixas F.K., Collares T., Nascimento R.J.M., Portela R.W., Azevedo V.A.C. (2020). NanH and PknG putative virulence factors as a recombinant subunit immunogen against Corynebacterium pseudotuberculosis infection in mice. Vaccine.

[13] Santana-Jorge K.T.O., Santos T.M., Tartaglia N.R., Aguiar E.L., Souza R.F.S., Mariutti R.B., Eberle R.J., Arni R.K., Portela R.W., Meyer R. (2016). Putative virulence factors of Corynebacterium pseudotuberculosis FRC41: Vaccine potential and protein expression. Microb. Cell Factories.

[14] de Pinho R.B., Silva M.T.D.O., Bezerra F.S.B., Borsuk S. (2021). Vaccines for caseous lymphadenitis: Up-to-date and forward-looking strategies. Appl. Microbiol. Biotechnol..

[15] Vale V.L.C., Silva M.D.C., De Souza A.P., Trindade S.C., De Moura-Costa L.F., Dos Santos-Lima E.K.N., Nascimento I.L.D.O., Cardoso H.S.P., Marques E.D.J., Paule B.J.A. (2016). Humoral and cellular immune responses in mice against secreted and somatic antigens from a Corynebacterium pseudotuberculosis attenuated strain: Immune response against a C. pseudotuberculosis strain. BMC Vet. Res..

[16] de Sá M.C.A., da Silva W.M., Rodrigues C.C.S., Rezende C.P., Marchioro S.B., Filho J.T.R.R., Sousa T.D.J., de Oliveira H.P., da Costa M.M., Figueiredo H.C.P. (2021). Comparative Proteomic Analyses Between Biofilm-Forming and Non-biofilm-Forming Strains of Corynebacterium pseudotuberculosis Isolated From Goats. Front. Vet. Sci..

[17] Rebouças M.F., Loureiro D., Bastos B., Moura-Costa L.F., Hanna S.A., Azevedo V., Meyer R., Portela R.W. (2013). Development of an indirect ELISA to detect Corynebacterium pseudotuberculosis specific antibodies in sheep employing T1 strain culture supernatant as antigen. Pesqui. Vet. Bras..

[18] McKean S.C., Davies J.K., Moore R.J. (2007). Expression of phospholipase D, the major virulence factor of Corynebacterium pseudotuberculosis, is regulated by multiple environmental factors and plays a role in macrophage death. Microbiology.

[19] Nascimento I.P., Leite L.C.C. (2012). Recombinant vaccines and the development of new vaccine strategies. Braz. J. Med. Biol. Res..

[20] Wu H.-C., Yeh P.-H., Hsueh K.-J., Yang W.-J., Chu C.-Y. (2018). Recombinant ApxIV protein enhances protective efficacy against *Actinobacilluspleuropneumoniae* in mice and pigs. J. Appl. Microbiol..

[21] Rosa M.C., Conrad N.L., Moraes C.M., Leite F.P.L. (2021). Immunogenicity of *Streptococcus equi* subsp. equi recombinant SeM protein and bacterin in mice. Pesqui. Vet. Bras..

[22] Hsueh K.-J., Cheng L.-T., Lee J.-W., Chung Y.-C., Chung W.-B., Chu C.-Y. (2017). Immunization with *Streptococcus suis* bacterin plus recombinant Sao protein in sows conveys passive immunity to their piglets. BMC Vet. Res..

[23] Khorasani A., Madadgar O., Soleimanjahi H., Keyvanfar H., Mahravani H. (2016). Evaluation of the efficacy of a new oil-based adjuvant ISA 61 VG FMD vaccine as a potential vaccine for cattle. Iran. J. Vet. Res..

[24] Zafra R., Buffoni L., Pérez-Caballero R., Molina-Hernández V., Ruiz-Campillo M.T., Pérez J., Martínez-Moreno Á., Moreno F.J.M. (2021). Efficacy of a multivalent vaccine against Fasciola hepatica infection in sheep. Vet. Res..

[25] Dorella F.A., Pacheco L.G., Seyffert N., Portela R.W., Meyer R., Miyoshi A., Azevedo V. (2009). Antigens of *Corynebacterium pseudotuberculosis* and prospects for vaccine development. Expert Rev. Vaccines.

[26] Corrêa J.I., Stocker A., Trindade S.C., Vale V., Brito T., Bastos B., Raynal J.T., de Miranda P.M., de Alcantara A.C., Freire S.M. (2018). In vivo and in vitro expression of five genes involved in Corynebacterium pseudotuberculosis virulence. AMB Express.

[27] Lopes Bastos B., Dias Portela R.W., Alves Dorella F., Ribeiro D., Seyffert N., de Paula Castro T.L., Miyoshi A., Costa Oliveira S., Meyer S., Azevedo V. (2012). Corynebacterium pseudotuberculosis: Immunological Responses in Animal Models and Zoonotic Potential. J. Clin. Cell. Immunol..

[28] Paule B.J.A., Azevedo V., Regis L.F., Carminati R., Bahia C.R., Vale V.L.C., Moura-Costa L.F., Freire S.M., Nascimento I., Schaer R. (2003). Experimental Corynebacterium pseudotuberculosis primary infection in goats: Kinetics of IgG and interferon-γ production, IgG avidity and antigen recognition by Western blotting. Vet. Immunol. Immunopathol..

[29] Bezerra F.S.B., Silva M.T.D.O., Rezende A.D.F.S., Lopes A.S., de Pinho R.B., Seixas F.K., Collares T.V., Portela R.W.D., Azevedo V.A.D.C., Borsuk S. (2021). Saponin-adjuvanted recombinant vaccines containing rCP00660, rCP09720 or rCP01850 proteins against Corynebacterium pseudotuberculosis infection in mice. Vaccine.

[30] Bezerra F.S.B., Rezende A.D.F.S., Silva M.T.D.O., Sena-Lopes Â., Roesch-Ely M., Henriques J.A.P., Padilha F.F., Azevedo V.A.C., Portela R.W.D., Seixas F.K. (2020). The combination of Brazilian red propolis and recombinant protein rCP01850 in the immunoprophylaxis of Corynebacterium pseudotuberculosis infection in mice. Microb. Pathog..

[31] Silva M.T.D.O., Bezerra F., de Pinho R.B., Begnini K.R., Seixas F.K., Collares T., Portela R., Azevedo V., Dellagostin O., Borsuk S. (2018). Association of Corynebacterium pseudotuberculosis recombinant proteins rCP09720 or rCP01850 with rPLD as immunogens in caseous lymphadenitis immunoprophylaxis. Vaccine.

